# Dimeth­yl(1,10-phenanthroline-κ^2^ *N*,*N*′)bis­(thio­cyanato-κ*N*)tin(IV)

**DOI:** 10.1107/S1600536812047691

**Published:** 2012-11-28

**Authors:** Ezzatollah Najafi, Mostafa M. Amini, Seik Weng Ng

**Affiliations:** a Department of Chemistry, General Campus, Shahid Beheshti University, Tehran 1983963113, Iran; b Department of Chemistry, University of Malaya, 50603 Kuala Lumpur, Malaysia; c Chemistry Department, Faculty of Science, King Abdulaziz University, PO Box 80203 Jeddah, Saudi Arabia

## Abstract

The Sn^IV^ atom in the title compound, [Sn(CH_3_)_2_(NCS)_2_(C_12_H_8_N_2_)], is located on a twofold rotation axis in a distorted octa­hedral enviroment. The methyl groups are *trans* to each other [C—Sn—C = 175.7 (3)°], whereas the thio­cyanate groups are *cis* to each other.

## Related literature

For dimethyl­tin dithio­thiocyanate, see: Britton (2006 ▶). For the 4,4′-bipyridine adduct, see: Najafi *et al.* (2011 ▶).
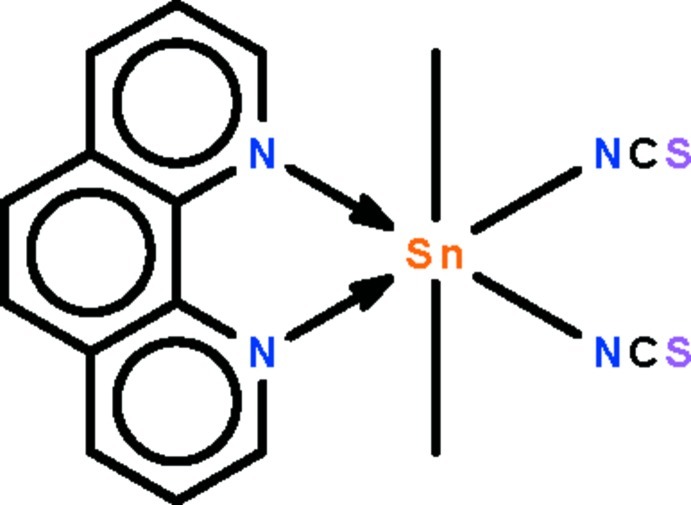


## Experimental

### 

#### Crystal data

[Sn(CH_3_)_2_(NCS)_2_(C_12_H_8_N_2_)]
*M* *_r_* = 445.12
Orthorhombic, 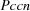

*a* = 6.8218 (7) Å
*b* = 12.9272 (13) Å
*c* = 20.746 (2) Å
*V* = 1829.5 (3) Å^3^
*Z* = 4
Mo *K*α radiation
μ = 1.63 mm^−1^
*T* = 295 K
0.30 × 0.15 × 0.05 mm

#### Data collection

Agilent SuperNova Dual diffractometer with an Atlas detector
Absorption correction: multi-scan (*CrysAlis PRO*; Agilent, 2012) ▶ *T*_min_ = 0.641, *T*_max_ = 0.923
10262 measured reflections
2116 independent reflections
1368 reflections with *I* > 2σ(*I*)
*R*_int_ = 0.055

#### Refinement

*R*[*F* ^2^ > 2σ(*F* ^2^)] = 0.039
*wR*(*F* ^2^) = 0.106
*S* = 1.01
2116 reflections
106 parameters
H-atom parameters constrained
Δρ_max_ = 0.51 e Å^−3^
Δρ_min_ = −0.63 e Å^−3^

### 

Data collection: *CrysAlis PRO* (Agilent, 2012 ▶); cell refinement: *CrysAlis PRO*; data reduction: *CrysAlis PRO*; program(s) used to solve structure: *SHELXS97* (Sheldrick, 2008 ▶); program(s) used to refine structure: *SHELXL97* (Sheldrick, 2008 ▶); molecular graphics: *X-SEED* (Barbour, 2001 ▶); software used to prepare material for publication: *publCIF* (Westrip, 2010 ▶).

## Supplementary Material

Crystal structure: contains datablock(s) global, I. DOI: 10.1107/S1600536812047691/bt6862sup1.cif

Structure factors: contains datablock(s) I. DOI: 10.1107/S1600536812047691/bt6862Isup2.hkl

Additional supplementary materials:  crystallographic information; 3D view; checkCIF report

## Figures and Tables

**Fig. 1.**
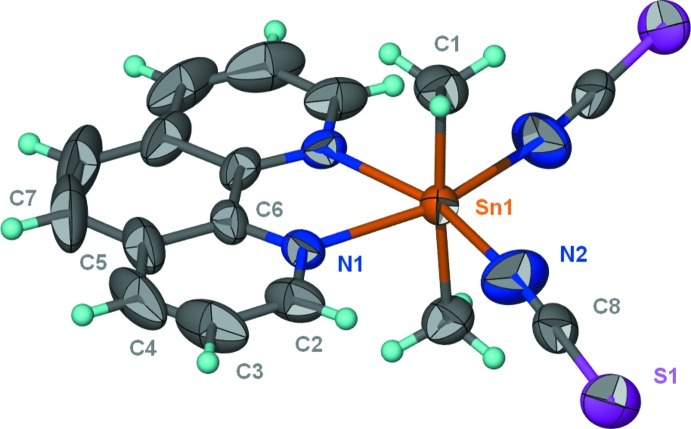
Thermal ellipsoid plot (Barbour, 2001) of (CH3)2Sn(NCS)2(C12H8N2) at the 50% probability level; hydrogen atoms are drawn as spheres of arbitrary radius.

## supplementary crystallographic information

### Comment

Few amine adducts of dimethyltin dithiocyanate, which exists as a weakly bridged polymeric chain (Britton, 2006), have been reported. The 4,4'-bipyridine adduct is polymeric (Najafi *et al.*, 2011). In the 1,10-phenanthroline adduct (Scheme I, Fig. 1), the Sn^IV^ atom is located on a twofold rotation axis in an octahedral enviroment. The methyl groups are *trans* to each other whereas the thiocyanate groups are *cis* to each other.

### Experimental

Dimethyltin dithiocyanate (0.27 g, 1 mmol) and 1,10-phenanthroline hydrate (0.19 g, 1 mmol) were loaded into a convection tube; the tube was filled with ethyl alcohol and kept at 333 K. Colorless crystals were collected from the side arm after several days.

### Refinement

Carbon-bound H-atoms were placed in calculated positions [C–H 0.93 to 0.96 Å, *U*_iso_(H) 1.2 to 1.5*U*_eq_(C)] and were included in the refinement in the riding model approximation.

### Crystal data

**Table:**

[Sn(CH_3_)_2_(NCS)_2_(C_12_H_8_N_2_)]	*F*(000) = 880
*M**_r_* = 445.12	*D*_x_ = 1.616 Mg m^−^^3^
Orthorhombic, *P**c**c**n*	Mo *K*α radiation, λ = 0.71073 Å
Hall symbol: -P 2ab 2ac	Cell parameters from 1757 reflections
*a* = 6.8218 (7) Å	θ = 3.2–27.5°
*b* = 12.9272 (13) Å	µ = 1.63 mm^−^^1^
*c* = 20.746 (2) Å	*T* = 295 K
*V* = 1829.5 (3) Å^3^	Prism, colorless
*Z* = 4	0.30 × 0.15 × 0.05 mm

### Data collection

**Table:**

Agilent SuperNova Dual diffractometer with an Atlas detector	2116 independent reflections
Radiation source: SuperNova (Mo) X-ray Source	1368 reflections with *I* > 2σ(*I*)
Mirror monochromator	*R*_int_ = 0.055
Detector resolution: 10.4041 pixels mm^-1^	θ_max_ = 27.6°, θ_min_ = 3.2°
ω scan	*h* = −6→8
Absorption correction: multi-scan (*CrysAlis PRO*; Agilent, 2012)	*k* = −16→16
*T*_min_ = 0.641, *T*_max_ = 0.923	*l* = −27→24
10262 measured reflections	

### Refinement

**Table:**

Refinement on *F*^2^	Secondary atom site location: difference Fourier map
Least-squares matrix: full	Hydrogen site location: inferred from neighbouring sites
*R*[*F*^2^ > 2σ(*F*^2^)] = 0.039	H-atom parameters constrained
*wR*(*F*^2^) = 0.106	*w* = 1/[σ^2^(*F*_o_^2^) + (0.0429*P*)^2^ + 0.5974*P*] where *P* = (*F*_o_^2^ + 2*F*_c_^2^)/3
*S* = 1.01	(Δ/σ)_max_ = 0.001
2116 reflections	Δρ_max_ = 0.51 e Å^−^^3^
106 parameters	Δρ_min_ = −0.63 e Å^−^^3^
0 restraints	Extinction correction: *SHELXL97* (Sheldrick, 2008), Fc^*^=kFc[1+0.001xFc^2^λ^3^/sin(2θ)]^-1/4^
Primary atom site location: structure-invariant direct methods	Extinction coefficient: 0.0024 (4)

### Fractional atomic coordinates and isotropic or equivalent isotropic displacement parameters (Å^2^)

**Table:**

	*x*	*y*	*z*	*U*_iso_*/*U*_eq_	
Sn1	0.7500	0.2500	0.243352 (18)	0.04816 (19)	
S1	0.3311 (2)	0.45584 (12)	0.09896 (7)	0.0957 (6)	
N1	0.5798 (5)	0.3037 (3)	0.33508 (16)	0.0515 (8)	
N2	0.5246 (7)	0.3231 (4)	0.1785 (2)	0.1073 (18)	
C1	0.5903 (7)	0.1114 (4)	0.2395 (2)	0.0746 (14)	
H1A	0.6337	0.0710	0.2034	0.112*	
H1B	0.4533	0.1267	0.2349	0.112*	
H1C	0.6109	0.0731	0.2786	0.112*	
C2	0.4146 (7)	0.3576 (4)	0.3337 (3)	0.0736 (14)	
H2	0.3600	0.3748	0.2941	0.088*	
C3	0.3208 (10)	0.3891 (5)	0.3898 (4)	0.107 (2)	
H3	0.2060	0.4276	0.3875	0.128*	
C4	0.3969 (12)	0.3635 (6)	0.4470 (4)	0.115 (3)	
H4	0.3327	0.3832	0.4846	0.138*	
C5	0.5721 (10)	0.3073 (5)	0.4510 (3)	0.0882 (18)	
C6	0.6600 (6)	0.2783 (3)	0.3924 (2)	0.0556 (11)	
C7	0.6688 (15)	0.2767 (9)	0.5097 (3)	0.142 (5)	
H7	0.6135	0.2956	0.5490	0.170*	
C8	0.4443 (7)	0.3778 (4)	0.1449 (2)	0.0644 (12)	

### Atomic displacement parameters (Å^2^)

**Table:**

	*U*^11^	*U*^22^	*U*^33^	*U*^12^	*U*^13^	*U*^23^
Sn1	0.0574 (3)	0.0459 (3)	0.0411 (3)	0.00503 (18)	0.000	0.000
S1	0.1188 (12)	0.0817 (11)	0.0866 (11)	0.0168 (9)	−0.0541 (9)	−0.0028 (8)
N1	0.056 (2)	0.043 (2)	0.055 (2)	0.0032 (17)	0.0090 (16)	−0.0054 (17)
N2	0.127 (4)	0.085 (4)	0.110 (4)	0.010 (3)	−0.058 (3)	0.019 (3)
C1	0.081 (4)	0.065 (3)	0.078 (4)	−0.010 (3)	−0.014 (2)	−0.009 (3)
C2	0.062 (3)	0.054 (3)	0.105 (4)	0.005 (2)	0.023 (3)	−0.010 (3)
C3	0.085 (4)	0.075 (4)	0.161 (7)	−0.002 (3)	0.061 (5)	−0.028 (5)
C4	0.137 (6)	0.096 (5)	0.113 (6)	−0.044 (5)	0.087 (5)	−0.050 (5)
C5	0.123 (5)	0.082 (4)	0.059 (3)	−0.039 (4)	0.038 (3)	−0.022 (3)
C6	0.071 (3)	0.052 (3)	0.044 (2)	−0.021 (2)	0.014 (2)	−0.008 (2)
C7	0.225 (15)	0.156 (12)	0.044 (3)	−0.092 (10)	0.035 (4)	−0.019 (4)
C8	0.071 (3)	0.068 (3)	0.054 (3)	−0.001 (3)	−0.015 (2)	−0.009 (2)

### Geometric parameters (Å, º)

**Table:**

Sn1—C1	2.098 (5)	C1—H1C	0.9600
Sn1—C1^i^	2.098 (5)	C2—C3	1.388 (8)
Sn1—N2^i^	2.251 (4)	C2—H2	0.9300
Sn1—N2	2.251 (4)	C3—C4	1.337 (10)
Sn1—N1	2.335 (3)	C3—H3	0.9300
Sn1—N1^i^	2.335 (3)	C4—C5	1.401 (9)
S1—C8	1.588 (5)	C4—H4	0.9300
N1—C2	1.326 (5)	C5—C6	1.405 (6)
N1—C6	1.350 (5)	C5—C7	1.442 (9)
N2—C8	1.134 (5)	C6—C6^i^	1.430 (9)
C1—H1A	0.9600	C7—C7^i^	1.31 (2)
C1—H1B	0.9600	C7—H7	0.9300
			
C1—Sn1—C1^i^	175.7 (3)	Sn1—C1—H1C	109.5
C1—Sn1—N2^i^	88.48 (19)	H1A—C1—H1C	109.5
C1^i^—Sn1—N2^i^	88.94 (18)	H1B—C1—H1C	109.5
C1—Sn1—N2	88.94 (18)	N1—C2—C3	121.9 (6)
C1^i^—Sn1—N2	88.48 (19)	N1—C2—H2	119.1
N2^i^—Sn1—N2	106.6 (3)	C3—C2—H2	119.1
C1—Sn1—N1	91.51 (16)	C4—C3—C2	119.5 (7)
C1^i^—Sn1—N1	92.01 (15)	C4—C3—H3	120.2
N2^i^—Sn1—N1	162.09 (16)	C2—C3—H3	120.2
N2—Sn1—N1	91.30 (17)	C3—C4—C5	120.7 (6)
C1—Sn1—N1^i^	92.01 (15)	C3—C4—H4	119.6
C1^i^—Sn1—N1^i^	91.51 (16)	C5—C4—H4	119.6
N2^i^—Sn1—N1^i^	91.30 (17)	C4—C5—C6	116.9 (6)
N2—Sn1—N1^i^	162.09 (16)	C4—C5—C7	125.6 (6)
N1—Sn1—N1^i^	70.80 (18)	C6—C5—C7	117.6 (7)
C2—N1—C6	119.4 (4)	N1—C6—C5	121.6 (5)
C2—N1—Sn1	124.2 (3)	N1—C6—C6^i^	118.2 (2)
C6—N1—Sn1	116.4 (3)	C5—C6—C6^i^	120.2 (4)
C8—N2—Sn1	163.6 (5)	C7^i^—C7—C5	122.2 (4)
Sn1—C1—H1A	109.5	C7^i^—C7—H7	118.9
Sn1—C1—H1B	109.5	C5—C7—H7	118.9
H1A—C1—H1B	109.5	N2—C8—S1	178.8 (5)
			
C1—Sn1—N1—C2	−89.4 (4)	Sn1—N1—C2—C3	−178.9 (4)
C1^i^—Sn1—N1—C2	88.1 (4)	N1—C2—C3—C4	−0.9 (9)
N2^i^—Sn1—N1—C2	−179.2 (5)	C2—C3—C4—C5	1.4 (10)
N2—Sn1—N1—C2	−0.5 (4)	C3—C4—C5—C6	−1.1 (9)
N1^i^—Sn1—N1—C2	179.0 (4)	C3—C4—C5—C7	179.2 (8)
C1—Sn1—N1—C6	91.6 (3)	C2—N1—C6—C5	0.2 (6)
C1^i^—Sn1—N1—C6	−90.9 (3)	Sn1—N1—C6—C5	179.3 (3)
N2^i^—Sn1—N1—C6	1.8 (6)	C2—N1—C6—C6^i^	−178.9 (4)
N2—Sn1—N1—C6	−179.5 (3)	Sn1—N1—C6—C6^i^	0.1 (6)
N1^i^—Sn1—N1—C6	0.0 (2)	C4—C5—C6—N1	0.3 (7)
C1—Sn1—N2—C8	−167.4 (17)	C7—C5—C6—N1	−180.0 (6)
C1^i^—Sn1—N2—C8	9.2 (17)	C4—C5—C6—C6^i^	179.4 (5)
N2^i^—Sn1—N2—C8	−79.3 (17)	C7—C5—C6—C6^i^	−0.8 (9)
N1—Sn1—N2—C8	101.1 (17)	C4—C5—C7—C7^i^	179.3 (13)
N1^i^—Sn1—N2—C8	99.4 (17)	C6—C5—C7—C7^i^	−0.5 (19)
C6—N1—C2—C3	0.0 (7)		

Symmetry code: (i) −*x*+3/2, −*y*+1/2, *z*.
